# Chloroplast Genomic Resource of *Paris* for Species Discrimination

**DOI:** 10.1038/s41598-017-02083-7

**Published:** 2017-06-13

**Authors:** Yun Song, Shaojun Wang, Yuanming Ding, Jin Xu, Ming Fu Li, Shuifang Zhu, Naizhong Chen

**Affiliations:** 1 Institute of Plant Quarantine, Chinese Academy of Inspection and Quarantine, Beijing, 100176 China; 2 Biological Germplasm Resources Identification Center of AQSIQ, Beijing, 100176 China; 3 Inspection and Quarantine Technology Center of Yunnan entry-exit inspection and Quarantine Bureau, Kunming, 650228 Yunnan China

## Abstract

*Paris* is famous in China for its medicinal value and has been included in the Chinese Pharmacopoeia. Inaccurate identification of these species could confound their effective exploration, conservation, and domestication. Due to the plasticity of the morphological characteristics, correct identification among *Paris* species remains problematic. In this regard, we report the complete chloroplast genome of *P*. *thibetica* and *P*. *rugosa* to develop highly variable molecular markers. Comparing three chloroplast genomes, we sought out the most variable regions to develop the best cpDNA barcodes for *Paris*. The size of *Paris* chloroplast genome ranged from 162,708 to 163,200 bp. A total of 134 genes comprising 81 protein coding genes, 45 tRNA genes and 8 rRNA genes were observed in all three chloroplast genomes. Eight rapidly evolving regions were detected, as well as the difference of simple sequence repeats (SSR) and repeat sequence. Two regions of the coding gene *ycf1*, *ycf1a* and *ycf1b*, evolved the quickest and were proposed as core barcodes for *Paris*. The complete chloroplast genome sequences provide more integrated and adequate information for better understanding the phylogenetic pattern and improving efficient discrimination during species identification.

## Introduction

The chloroplasts are photosynthetic organelles that provide energy to green plants. In angiosperms, most chloroplast genomes are circular, double-stranded DNA, containing a pair of inverted repeats (IRs), one large single-copy region (LSC) and one small single copy region (SSC)^1, 2^. Most chloroplast genomes are ranging from 120–160 kb in length and highly conserved in gene content and order^3, 4^. Owing to being haploid, maternal inheritance, and highly conservation in gene content and genome structure, the chloroplast genomes are valuable sources for exploring useful DNA markers for species identification, evolutionary studies and phylogenetic relationships among plant species^5–7^. The advance of high-throughput sequencing technologies has facilitated rapid progress of chloroplast genomics due to time-saving and low-cost advantages^8^. The number of chloroplast genomes of land plants released in the National Center for Biotechnology Information (NCBI) has risen to 1,011 (accessed at October 31, 2016).

The genus *Paris* (Melanthiaceae: Parideae)^9, 10^ consists of about 24 species of perennial herbs distributed in the temperate regions from Europe to eastern Asia, 22 species (12 endemic) were chiefly in China. The rhizomes of many *Paris* species are used in traditional Chinese medicine for more than 2000 years in China, owing to their analgesic and anti-coagulant properties, most notably as an ingredient of Yunnan Baiyao^11^. However, over-exploitation for economic purposes is pushing these species to the brink of extinction. The *Paris* genus is listed as exit-prohibited species by Environmental Protection Agency. So there is an urgent need to develop conservation strategies to prevent losses of species resources through the characterization of its genomic information and genetic structure.

Because of their medicine value, *Paris* species has been the subject of taxonomic studies and, particularly, species identification^12, 13^. However, so far, there are no efficient methods for identifying the species of *Paris*. Traditionally, the taxonomy and species identification of the genus *Paris* are based on the morphological traits, but the plasticity of its morphological characteristics made the classification of *Paris* very complicated, most *Paris* species have abundant intraspecific variations in morphology and chemical composition^12, 14, 15^.

Molecular methods, such as molecular marker techniques and DNA barcoding, provide effective information for taxonomy and species identification. In the past decades, the applications of diverse molecular techniques have gained increasing importance in resolving taxonomy and species identification questions. However, at the species level, the reported candidate barcoding sequences still have difficulties in the identification of *Paris* species. Analysis based on plastid genomic markers (*psbA-tmH*, *rpoB*, *rpoCl*, *rbcL*, *matK*) and nuclear gene ITS2 suggested that ITS2 can only discriminate *P*. *polyphylla* var. *yunnanensis* from *P*. *polyphylla* var. *chinensis* ^16–19^. Ji *et al*. tested the generic and infrageneric circumscription of *Paris* with nuclear ITS and plastid *psbA-trnH*, *trnL-trnF* DNA sequence data and supported the classification of *Paris* as a single genus, but the delimitation of species still remained unresolved^12^. All these studies have provided valuable insights for an initial molecular-based identification of *Paris*, but there were too little variations in those chloroplast genomic markers to address the issues of species discrimination.

Here, we sequenced and analyzed the chloroplast genome of *P*. *rugosa* and *P*. *thibetica* using the next-generation sequencing platform. Our aim was to retrieve valuable chloroplast molecular markers by comparing the chloroplast genomes among these two and recently published chloroplast genomes of *Paris*. Our second objective was to investigate global structural patterns of *Paris* chloroplast genomes and to examine variations of simple sequence repeat (SSRs) and repeat sequences among *Paris* chloroplast genomes. We believe that these types of resources will be useful for species-level discrimination and avoid confounding effective exploration, conservation, and domestication for *Paris* species.

## Results

### Genome Assembly and Features

**Figure 1:**
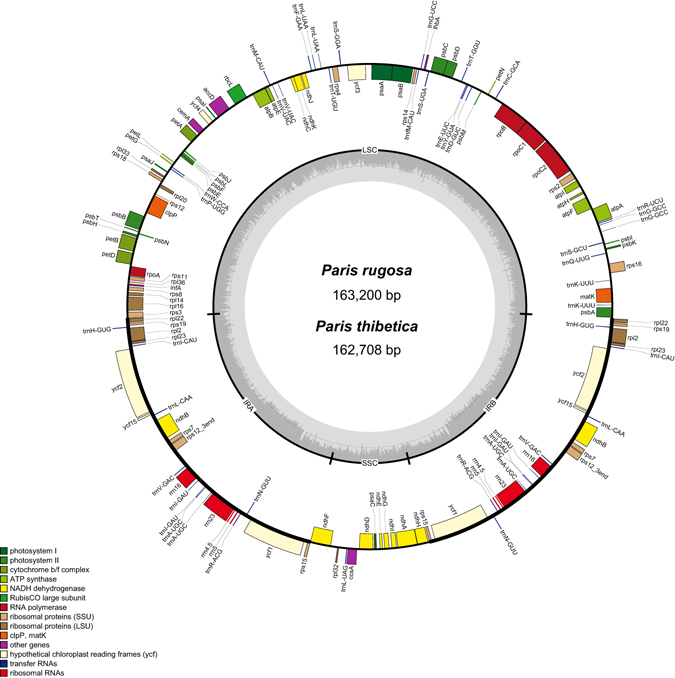
Gene map of *Paris* Chloroplast genome. The genes inside and outside of the circle are transcribed in the clockwise and counterclockwise directions, respectively. Genes belonging to different functional groups are shown in different colors. The thick lines indicate the extent of the inverted repeats (IRa and IRb) that separate the genomes into small single-copy (SSC) and large single-copy (LSC) regions.

We sequenced the complete chloroplast genome of two *Paris* species, *P*. *rugosa* and *P*. *thibetica* (Fig. 1). In total, 10,380,007 (*P*. *rugosa*) and 26,745,248 (*P*. *thibetica*) raw data reads were generated. Out of those, 401,240 and 297,202 reads were identified as the chloroplast genome sequences for *P*. *rugosa* and *P*. *thibetica*, respectively (Table 1). Chloroplast genomes showed a typical quadripartite structure, consisting of a pair of IRs (32,884–33,144 bp) separated by the LSC (84,010–84,108 bp) and SSC (12,854–12,984 bp) regions (Fig. 1 and Table 1). The chloroplast genome of *P*. *rugosa* (GenBank accession no. KY247142), with a length of 163,200 bp, was 492 bp larger than that of *P*. *thibetica* (GenBank accession no. KY247143), 210 bp larger than that of *P*. *polyphylla* var. *yunnanensis* (GenBank accession no. KT805945) published in our previous paper.

**Table 1:** Comparison of feature of *Paris rugose*, *Paris thibetica*, *Paris polyphylla* var. *yunnanensis*.

Species	*Paris rugosa*	*Paris thibetica*	*Paris polyphylla* var. *yunnanensis*
Accession number	KY247142	KY247143	KT805945
Genome size (bp)	163200	162708	162990
LSC (bp)	84058	84010	84108
SSC (bp)	12854	12930	12984
IRs (bp)	33144	32884	32949
Number of protein-coding genes^1^	81(9)	81(9)	81(9)
Number of tRNAs genes^1^	45(8)	45(8)	45(8)
Nubmer of rRNAs genes^1^	8(4)	8(4)	8(4)
GC content (%)	37.1	37.2	37.1
Raw data read number	10380007	26745248	/
Mapped read number	401240	297202	/
Chloroplast coverage(X)	368	373	/

^1^The numbers in parenthesis indicate the genes duplicated in the IR regions.

Three *Paris* genomes identically harbored 113 different genes arranged in the same order, including 72 protein-coding genes, 37 tRNA genes and 4 rRNA genes. All these three genomes have rich AT content with an overall purine content ranging from 62. 8% to 62.9% (Table 1).

### SSR and Repetitive Sequence Statistics

**Figure 2:**
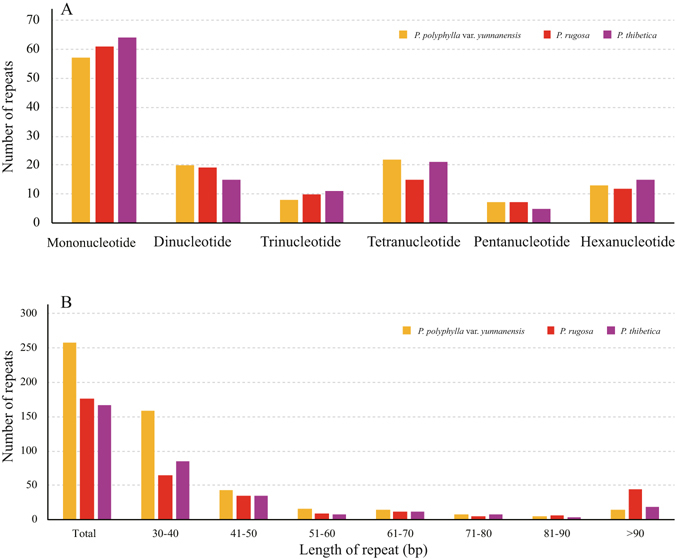
Analysis of repeated sequences in the three *Paris* chloroplast genomes. (**A**) Frequency of simple sequence repeats (SSRs) by MISA. (**B**) Frequency of repeat sequences determined by REPuter.

SSRs are repeated DNA sequences consisting of tandem repeats 1–10 bp in length per unit distributed throughout the genome (Fig. 2A). The total number of SSRs was 127 in *P*. *polyphylla* var. *yunnanensis*, 124 in *P*. *rugosa* and 131 in *P*. *thibetica* (Supplementary Table S3). The majority type of SSR in all species was mononucleotide, with 57 in *P*. *polyphylla* var. *yunnanensis*, 61 in *P*. *rugosa* and 64 in *P*. *thibetica* (Supplementary Table S1).

Repeat sequences with repeat unit longer than 30 bp and sequence identity greater than 90% were analyzed (Fig. 2B). *P*. *polyphylla* var. *yunnanensis* contained 258 repeats, of these, 159 repeats were 30–40 bp long, 85 repeats were 40–90 bp long, and 14 repeats were longer than 90 bp. *P*. *rugosa* contained 176 repeats, of these, 65 repeats were 30–40 bp long, 67 repeats were 40–90 bp long, and 44 repeats were longer than 90 bp. *P*. *thibetica* contained 167 repeats, of these, 85 repeats were 30–40 bp long, 64 repeats were 40–90 bp long, and 18 repeats were longer than 90 bp (Fig. 2B, Supplementary Table S4).

### Divergent Hotspots in *Paris* Chloroplast Genome

**Figure 3:**
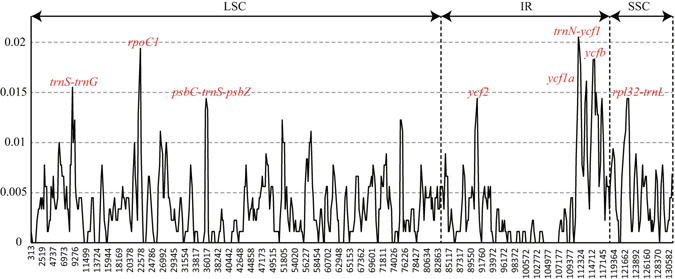
Sliding window analysis of the whole chloroplast genome of three *Paris* species. (window length: 600 bp, step size: 200 bp). X-axis: position of the midpoint of a window, Y-axis: nucleotide diversity of each window.

A total of 902 SNPs were detected among three *Paris* species. To clarify the sequence divergence level, the nucleotide variability values within 600 bp in all three chloroplast genomes were calculated with DnaSP 5.0 software. The values ranged from 0 to 0.02056 with a mean of 0.00375, revealing the slight differences among the genomes. However, eight highly variable loci with higher Pi values (Pi > 0.0087) were precisely located (Fig. 3). These regions included *trnS-trnG*, *rpoC1*, *psbC-trnS-psbZ*, *ycf2*, *ycf1a*, *trnN-ycf1*, *ycf1b*, *rpl32-trnL*, of which three loci lie in the LSC region, four in the IR region, and one in the SSC region (Fig. 3).

### DNA barcoding of *Paris*

*TrnN-ycf1* had some more indels and poly structure and the primers did not work well, so we gave it up in the following analyses. The variability of seven developed regions were tested together with three conventional candidate DNA barcodes (*matK*, *rbcL* and *trnH-psbA*) using 19 samples of *Paris* species. Features of ten barcode data set were shown in Table 2. There are only six variable sites of the *trnH-psbA* region, showing the lowest level of variability (0.68%). The variability of the *ycf1a* region was the highest (7.72%), followed by *ycf1b* region (6.47%), *trnS-trnG* region (6.25%), and *rpl32-trnL* (5.25%).

**Table 2:** Variability of the seven new markers and universal chloroplast DNA barcode in *Paris*.

Markers	Region	Length	Variable sites		Information sites		Nucleotide diversity	Discrimination success (%) based on Distance method
			Numbers	%	Numbers	%		
*trnS-trnG*	LSC	1279	80	6.25%	38	2.97%	0.00602	47.37%
*rpoC1*	LSC	999	24	2.40%	17	1.70%	0.00589	15.79%
*psbC-trnS-psbZ*	LSC	1035	12	1.16%	8	0.77%	0.00226	42.11%
*ycf2*	IR	940	29	3.09%	21	2.23%	0.00846	36.84%
*ycf1a*	IR	1140	88	7.72%	57	5.00%	0.01826	52.63%
*ycfb*	IR	556	36	6.47%	23	4.14%	0.01442	31.58%
*rpl32-trnL*	SSC	1010	53	5.25%	31	3.07%	0.00226	47.37%
*ycf1a* + *ycf1b*	IR	1696	124	7.31%	80	4.72%	0.01737	89.47%
*matK*	LSC	734	7	0.95%	4	0.54%	0.00197	21.05%
*rbcL*	LSC	602	10	1.66%	8	1.33%	0.00456	26.32%
*trnH-psbA*	LSC	876	6	0.68%	5	0.57%	0.00172	5.26%
*matK* + *rbcL* + *trnH-psbA*	LSC	2212	23	1.04%	17	0.77%	0.00256	42.11%

**Figure 4:**
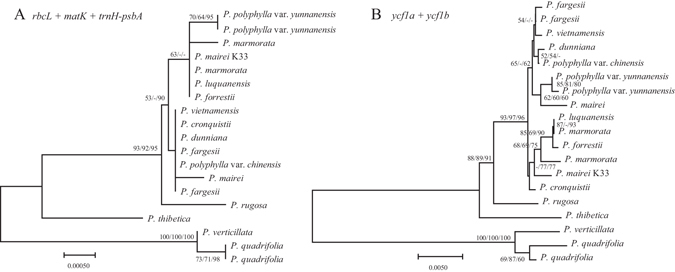
NJ tree for *Paris* using the *rbcL* + *matK* + *trnH-psbA* (**A**) and *ycf1a* + *ycf1b* (**B**) DNA barcode combination. NJ topology shown with NJ/MP/ML bootstrap support values were listed at each node.

In the single-barcode analysis using distance method, the lowest discriminatory power was found for *trnH-psbA* (5.26%), followed by *rpoC1*(15.79%) and *matK* (21.05%), while *ycf1a* (52.63%) provided the highest discrimination rate. Combining *matK* + *rbcL* + *trnH-psbA*, the discrimination rate was still relatively low (42.11%). According to the single barcode discrimination power, the combination of *ycf1a* + *ycf1b* presented a higher discrimination rate (89.47%). The tree based method had the same results (Fig. 4 and Supplementary Fig. 1).

## Discussion

### Chloroplast Genome of *Paris*

Recently, more and more taxonomists have focused on chloroplast genome to investigate phylogeny relationship of related species. For example, the chloroplast genome of three species of Veroniceae^20^ and four species of *Tila* ^21^ were used for plant phylogenetic analysis. In this study, the complete plastid genome sequences of three *Paris* species were compared and the results showed that the gene structures, contents and arrangement were conserved. The size of *P*. *thibetica*, *P*. *rugosa* and *P*. *polyphylla* var. *yunnanensis* chloroplast genome ranged from 162,708 to 163,200 bp, nevertheless, the three *Paris* species had the same protein-coding genes, tRNAs and rRNAs. The length variations among *Paris* chloroplast genomes may result from the length of spacer and intron.

Compared with other Melanthiaceae chloroplast genomes, IR regions extended into *rps15* gene in *Paris* and genome size is ~7 kb longer than *Trillium* ^22^. The IR/SC junction position changes may be caused by contraction or expansion of IR region, which is a common evolutionary phenomenon in plants^23^.

Larger and more complex repeat sequences may play an important role in the rearrangement of chloroplast genomes and sequence divergence^23^. In the three *Paris* chloroplast genomes, we found numerous repeated sequences particularly in the intergenic spacer regions and the length of repeated sequences ranged from 30 to 284 bp, similar to those reported in other angiosperm linages^24, 25^.

Previously, SSRs have been described as a major tool to unravel genome polymorphism across species, ecological and evolutionary studies^4, 26^. In three *Paris* chloroplast genomes, the most abundant SSR pattern was found to be stretches of mononucleotides (A/T) (Fig. 2A). More interestingly, the cpSSRs were only observed in the non-coding region^27, 28^. Because the chloroplast genome sequences are highly conserved among *Paris*, microsatellite sites for chloroplast genomes are transferable across species. The cpSSRs of three *Paris* species in our study are expected to be useful for the analysis of genetic diversity in *Paris*.

### DNA barcode for *Paris*

DNA barcoding has been largely used as a new biological tool to facilitate accurate species identification^29^. The ideal DNA barcode would be a single locus that could be universally amplified and sequenced for a broad range of taxa, be easily aligned over large phylogenetic distances, and provide sufficient variation to reliably distinguish closely related species^30^. Unfortunately, the candidate barcodes *matK* and *rbcL*, as a “core” plant barcode, often have limited resolutions at species level. In this study, combining *matK*, *rbcL* and *trnH-psbA* only less than half of samples were successfully identified (Table 2). Therefore, searching for an effective barcode with high evolutionary rates is very important for specific group, such as *Paris*.

Chloroplast genome is endemic to plants. Therefore, chloroplast DNA barcodes are of primary choices. The “hotspot” regions which cluster more SNP and indel mutations create the highly variable regions in the chloroplast genome. In this study, we identified eight highly variable barcode including *trnS-trnG*, *rpoC1*, *psbC-trnS-psbZ*, *ycf2*, *ycf1a*, *trnN-ycf1*, *ycf1b*, *rpl32-trnL* (Fig. 3). The coding gene *ycf1*, *trnS-trnG*, *rpoC1* and *rpl32-trnL* were the focus in previous studies to investigate sequence variation and phylogenetic analysis in angiosperms^4, 31, 32^.

The poor performance of three commonly used barcodes *rbcL*, *trnH-psbA*, and *matK* in resolving *Paris* species indicates that additional barcodes should be exploited for this complex group. The *ycf1a* and *ycf1b* regions can be used as a starting point to identify *Paris* and relative species because they are certainly the most promising sequences to accomplish DNA barcode objectives in closely related species up to now. *ycf1* encodes a protein of approximately 1,800 amino acids, as the second largest gene in the chloroplast genome^33^. Because *ycf1* is too long and too variable to permit the design of universal primers^31^, it has received little attention for DNA barcoding at low taxonomy, but *ycf1*, especially *ycf1a* and *ycf1b* may be the best barcodes at present as specific barcodes for *Paris* (Fig. 4 and Table 2).

The chloroplast genomes provide sufficient genetic information for species identification. In this study, we identified variable markers in the chloroplast genome for accurate *Paris* species identification and developed SSRs for further evolutionary studies. Such strategy to invent species-specific molecular markers was an effective approach that it will increase the efficiency and feasibility of species identification and population-based studies of *Paris* considering the characteristics of the chloroplast genomes.

## Materials and Methods

### Chloroplast Genome Sequencing

Fresh leaves were collected from Lushui, Yunnan province in South China and were identified based on morphology. Total genomic DNA was isolated from fresh leaves using the DNeasy Plant MiniKit (Qiagen, CA, USA). DNA and voucher specimens of sampled species were deposited in the herbarium of Chinese Academy of Inspection and Quarantine. DNA was sheared by nebulization with compressed nitrogen gas, yielding fragments of 500 bp in length. Paired-end libraries were prepared with the Mate Pair Library Preparation Kit (Illumina, San Diego, California, USA) in accordance with the manufacturer’s instructions. Whole genome sequences were executed using Illumina Hiseq 4000 Genome Analyzer.

### Chloroplast Genome Assemblage and Annotation

For both two species, the high-throughput sequencing data were quality-controlled and assembled using SPAdes 3.6.1^34^. The assembled sequences of the chloroplast genome were selected using the Blast program^35^. The contigs of the chloroplast genome were assembled using Sequencher 4.10 with default parameters and the gaps between contigs were linked by amplification with PCR-based conventional Sanger sequencing using ABI 3730. The specific primers were designed based on the flanking sequences to bridge the gaps. After that, all reads were mapped to the assembled chloroplast genome sequence using Geneious 8.1^36^ to avoid assembly errors and proofread the contig is correct. Finally, we obtained two Paris high quality complete chloroplasts genome sequences. The assembled genomes were annotated using the Dual Organellar Genome Annotator (DOGMA)^37^. The circle maps of the two species were drawn using GenomeVx^38^.

### Repeat Sequence Analysis

Perl script MISA (MIcroSAtellite identification tool, http://pgrc.ipk-gatersleben.de/misa/) was used to search for simple sequence repeat (SSRs or microsatellites) loci in the chloroplast genomes. Tandem repeats of 1–6 nucleotides were considered as microsatellites. The minimum numbers of repeats were set to 10, 6, 5, 5, 5 and 5 for mono-, di-, tri-, tetra-, penta-, and hexa-nucleotides, respectively. REPuter was used to find tandem, dispersed, and palindromic repeats, with a minimum repeat size of 30 bp and a sequence identity greater than 90%^39^.

### Divergent Hotspots Identification

The three completed chloroplast genome sequences (*P*. *polyphylla* var. *yunnanensis*, *P*. *rugose*, *P*. *thibetica*) were aligned using MAFFT^40^ and were manually adjusted using Se-Al 2.0^41^. To analyze nucleotide diversity (Pi), we conducted a sliding window analysis using DnaSP version 5 software^42^. The window length was set to 600 base pairs and the step size was set as 200 base pairs.

### Highly Variable Barcode Acquisition

We collected 6 *Paris* species to test the barcodes designed in this study (Supplementary Table S1). The primers for amplifying the highly variable regions were designed using FastPCR (Supplementary Table S2). The primers for amplifying and sequencing the control markers of *rbcL*, *matK* and *trnH-psbA* were the same as previous studies^33^. The same DNA sequences of another 11 *Paris* species were downloaded from GenBank^43^.

The PCR amplifications were performed in a final volume of 25 μL containing 1× PCR buffer (with Mg^2+^), 0.25 mmol/L each dNTP, 0.25 μmol/L each primer, 1.25 U Taq polymerase, and 20–30 ng DNA. The PCR program started at 94 °C for 4 min, followed by 34 cycles of 30 s at 94 °C, 40 s at 52 °C, and 1 min at 72 °C, and ended with a final extension of 10 min at 72 °C. Both of the strands were sequenced on ABI Prism 3730xl (Applied Biosystems, Foster City, U.S.A.) following the manufacturer’s protocols.

### DNA Barcoding Analysis

We evaluated the hypervariable barcodes and compared with the chloroplast genes *rbcL*, *matK* and *trnH-psbA* using two different methods. Firstly, the distance method was applied via the function nearNeighbour of SPIDER^44^. Species discrimination was considered successful if the closest K2P distance for all of the individuals of a given species belonged to only one conspecific individual. Secondly, a tree-based method was used to assess whether sequences in our data sets form species specific clusters. Neighbour-joining (NJ) trees were constructed for each individual barcode and their combinations by MEGA 6 based on a K2P distance^45^. Maximum likelihood (ML) analyses were performed using RAxML 8.0 with the GTR+G model^46^. Maximum parsimony (MP) trees were analyzed with PAUP* v4b10 program^47^. Relative support for the branches of the NJ, ML and MP trees were assessed via 1000 bootstrap replicates.

## Electronic supplementary material

Supplementary tables and figures
