# Supplementary material for: Chloroplast Genomic Resource of Paris for Species Discrimination
Source: Sci Rep. 2017 Jun 13;7:3427. doi: 10.1038/s41598-017-02083-7 (PMC5469780; doi:10.1038/s41598-017-02083-7)

Chloroplast Genomic Resource of *Paris* for Species Discrimination

Yun Song [1,2], Shaojun Wang [3], Yuanming Ding [3], Jin Xu [1,2], MingFu Li [1,2], Shuifang Zhu [1], Naizhong Chen [1,2*]

[1]Institute of Plant Quarantine, Chinese Academy of Inspection and Quarantine, Beijing 100176, China.

[2] Biological Germplasm Resources Identification Center of AQSIQ, Beijing 100176, China.

[3] Inspection and Quarantine Technology Center of Yunnan entry-exit inspection and Quarantine Bureau, Kunming 650228, Yunnan, China.

[*] Corresponding author: chennz@263.net.cn

Table S1. GenBank accessions for samples of *Pairs*.

Species	Vouchers	*trnS-trnG*	*rpoC1*	*psbC-trnS-psbZ*	*ycf2*	*ycf1a*	*ycf1b*	*rpl32-trnL*	*matK*	*rbcL*	*trnH-psbA*
P. cronquisitii	/	KX784041	KX784041	KX784041	KX784041	KX784041	KX784041	KX784041	KX784041	KX784041	KX784041
P. dunniana	/	KX784042	KX784042	KX784042	KX784042	KX784042	KX784042	KX784042	KX784042	KX784042	KX784042
P. fargesii	/	KX784043	KX784043	KX784043	KX784043	KX784043	KX784043	KX784043	KX784043	KX784043	KX784043
P. fargesii	K14	KY851338	KY851328	KY851363	KY851353	KY851343	KY851348	KY851373	KY851358	KY851368	KY851333
P. forrestii	/	KX784044	KX784044	KX784044	KX784044	KX784044	KX784044	KX784044	KX784044	KX784044	KX784044
P. luquanensis	/	KX784045	KX784045	KX784045	KX784045	KX784045	KX784045	KX784045	KX784045	KX784045	KX784045
P. mairei	/	KX784046	KX784046	KX784046	KX784046	KX784046	KX784046	KX784046	KX784046	KX784046	KX784046
P. mairei	K33	KY851339	KY851329	KY851364	KY851354	KY851344	KY851349	KY851374	KY851359	KY851369	KY851334
P. marmorata	/	KX784047	KX784047	KX784047	KX784047	KX784047	KX784047	KX784047	KX784047	KX784047	KX784047
P. marmorata	K36	KY851340	KY851330	KY851365	KY851355	KY851345	KY851350	KY851375	KY851360	KY851370	KY851335
P. polyphylla var. *chinensis*	/	KX784048	KX784048	KX784048	KX784048	KX784048	KX784048	KX784048	KX784048	KX784048	KX784048
P. polyphylla var. *yunnanensis*	A40	KT805945	KT805945	KT805945	KT805945	KT805945	KT805945	KT805945	KT805945	KT805945	KT805945
P. polyphylla var. *yunnanensis*	/	KX784049	KX784049	KX784049	KX784049	KX784049	KX784049	KX784049	KX784049	KX784049	KX784049
P. quadrifolia	/	KX784051	KX784051	KX784051	KX784051	KX784051	KX784051	KX784051	KX784051	KX784051	KX784051
P. quadrifolia	K15	KY851341	KY851331	KY851366	KY851356	KY851346	KY851351	KY851376	KY851361	KY851371	KY851336
P. rugosa	K32	KY247142	KY247142	KY247142	KY247142	KY247142	KY247142	KY247142	KY247142	KY247142	KY247142
P. thibetica	K35	KY247143	KY247143	KY247143	KY247143	KY247143	KY247143	KY247143	KY247143	KY247143	KY247143
P. verticillata	K16	KY851342	KY851332	KY851367	KY851357	KY851347	KY851352	KY851377	KY851362	KY851372	KY851337
P. vietnamensis	/	KX784050	KX784050	KX784050	KX784050	KX784050	KX784050	KX784050	KX784050	KX784050	KX784050

Table S2. Primers for amplifying and sequencing 7 highly variable regions.

	Forward primer	Reverse primer
Locus	Sequence 5' to 3'	Sequence 5' to 3'
psbC-trnS-psbZ	CAAGGACCCACTGGTTTAGGTA	CCAAGCTACCGACAAATAAACTA
rpl32-trnL	TCTTTAGCTAAATCTGTTTCCAC	TACATTATAGGATCCATTATGTC
rpoC1	ATGAACAGCCATTTGATCCCCATC	CCGGAAATACAGTATCCCGCTT
trnS-trnG	TGATGTGACACGCGAAGTTGAAGTA	ATCAAGATAGCATCAGAATCATGA
ycf1a	AACTATTGAAATTATTATCACTA	TAGCAGACGGGATTCAGATAAAG
ycf1b	CAGTCTAAGTAGGAAACAATAC	CTTGGACCAATACGCATGTTGGC
ycf2	ATTGAATCGATTCCTTTTAATG	AAGTATCATGATCATACAATAAGA

Table S3.Simple sequence repeats of three *Paris* species

Species	Unit size	Number of SSRs
P. polyphylla var. yunnanensis	mononuleotide	57
	dinucleotide	20
	trinucleotide	8
	tetranucleotide	22
	pentanucleotide	7
	hexanucleotide	13
P. rugosa	mononuleotide	61
	dinucleotide	19
	trinucleotide	10
	tetranucleotide	15
	pentanucleotide	7
	hexanucleotide	12
P. thibetica	mononuleotide	64
	dinucleotide	15
	trinucleotide	11
	tetranucleotide	21
	pentanucleotide	5
	hexanucleotide	15

Table S4.Repeat sequence of three *Paris* species

Species	Repeat size	Number of repeats
P. polyphylla var. yunnanensis	30-40	159
	41-50	43
	51-60	16
	61-70	14
	71-80	7
	81-90	5
	>90	14
P. rugosa	30-40	65
	41-50	35
	51-60	9
	61-70	12
	71-80	5
	81-90	6
	>90	44
P. thibetica	30-40	85
	41-50	35
	51-60	8
	61-70	11
	71-80	7
	81-90	3
	>90	18

Figure S1. NJ trees of *Paris* species based on 7 chloroplast barcodes, showing the resolutions of the loci in the group. The figures above the lines are the bootstrap values for the clades.

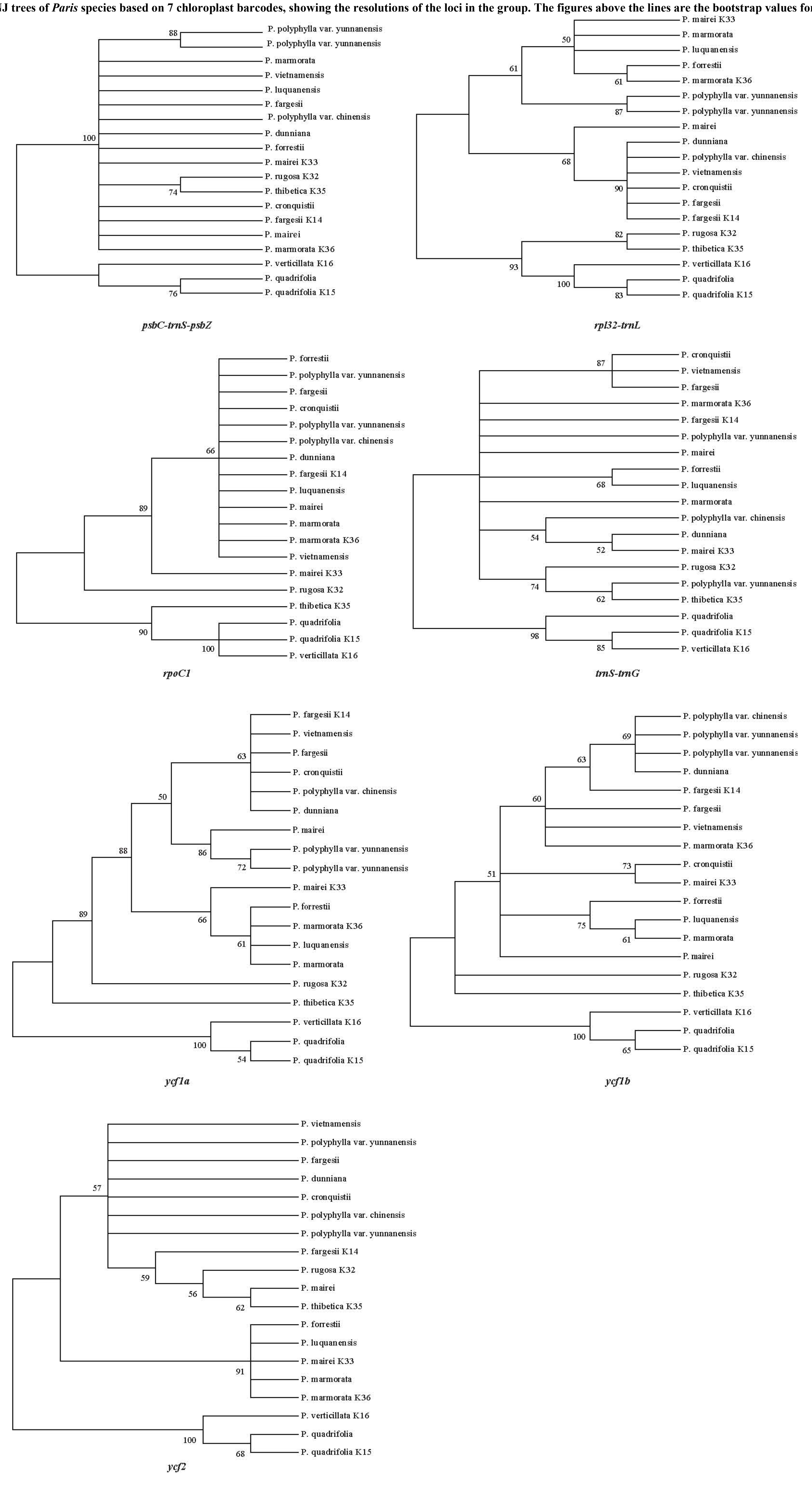

Supplement: Supplementary file 1 — Supplementary tables and figures [file 41598_2017_2083_MOESM1_ESM.pdf]
